# From markers to mechanisms: a comprehensive review of major genes and quantitative trait loci shaping the modern sheep (*Ovis aries*)

**DOI:** 10.3389/fgene.2026.1865980

**Published:** 2026-08-05

**Authors:** Mostafa Ghaderi-Zefrehei, Effat Nasre Esfahani, Hassan Amini Pozveh, Mohammadreza Hashemi, Parisa Dolati, Sonia Zakizadeh, Maryam Montazeri, Mustafa Muhaghegh Dolatabady, Ikhide Godwin Imumorin, Mohammad Hossein Banabazi

**Affiliations:** 1 Department of Animal Science, Faculty of Agriculture, Yasouj University, Yasouj, Iran; 2 Department of Agriculture, Payame Noor University, Tehran, Iran; 3 Department of Animal Science, Faculty of Agriculture Sciences, Bu-Ali Sina University, Hamedan, Iran; 4 Department of Genetics and Animal Breeding, Faculty of Agricultural Sciences, University of Tehran, Karaj, Iran; 5 Genetics Research Center, University of Social Welfare and Rehabilitation Sciences, Tehran, Iran; 6 Animal Science Research Institute of Iran, Agricultural Research, Education and Extension Organization (AREEO), Karaj, Iran; 7 Department of Animal Science, Faculty of Agricultural Sciences, University of Guilan, Rasht, Iran; 8 California State University Biotechnology Program (CSUBIOTECH), College of Science, San Diego State University, San Diego, CA, United States; 9 Department of Animal Biosciences (HBIO), Swedish University of Agricultural Sciences (SLU), Uppsala, Sweden; 10 Department of Biotechnology, Animal Science Research Institute of IRAN (ASRI), Agricultural Research, Education and Extension Organization (AREEO), Karaj, Iran

**Keywords:** disease resistance, genomic selection, growth traits, GWAS, linkage mapping, multi-omics, reproduction, RNA-sequencing

## Abstract

One of the most important agricultural industries in the world is sheep (*Ovis aries*), considering essential resources such as meat, milk, and wool. Genetic and genomics innovations have significantly improved the efficiency and sustainability of sheep production, which requires a thorough knowledge of the genetic basis of economically relevant traits that are controlled. This is a comprehensive review of the historical development of sheep genetics, from basic stages in the use of microsatellite markers in linkage mapping to the current environment of high-throughput genotyping methods, whole-genome sequencing, and GS (Genomic Selection), which collectively form the basis of contemporary improvement programs. The review is organized by major trait categories, which include growth and body composition, reproduction and fertility, wool quality, and disease resistance. The major genes and QTL (Quantitative trait loci) of each category are defined and described, as well as their biological roles, molecular mechanisms, and their usage in modern breeding solutions are discussed. The most important methodologies that enable these discoveries are elucidated, including linkage analysis, GWAS (Genome-wide association studies), RNA-sequencing (RNA-Seq), and newly emerging multi-omics approaches. Comparative studies also show that there are conserved genetic processes and structures in various sheep breeds and other livestock species, specifically cattle, which indicates species-specific and common regulatory pathways. Rather than claiming to be an exhaustive historical record, this narrative review synthesizes major advances in sheep genetic mapping, functional genomics, and GS relevant to economically important traits. By critically evaluating the strength of evidence, mapping resolution, and practical breeding applications, this work aims to provide researchers, breeders, and students with a balanced, evidence-based resource to guide future genomic improvements in sustainable sheep production.

## 1 Introduction

Sheep domestication stands as one of the important milestones in the transition to agricultural societies, providing essential resources such as meat, milk, and hides (Kaptan et al., 2024; Chessa et al., 2009). While the traditional domestication window is often cited as 10,000–8,000 BCE in Southwest Asia, contemporary evidence suggests a more geographically diverse and temporally protracted process, occurring as early as 12,000 years ago during the early Holocene (Li and Salehian Dehkordi, 2026b). Archaeological records from key Neolithic localities, including the Zagros mountains and the central Anatolian site of Aşıklı Höyük, demonstrate that sheep management was well-established by 9,000–8,000 BCE (Li and Salehian Dehkordi, 2026b). This complex evolutionary trajectory is further elucidated by recent paleogenomic research, which indicates that major migration waves occurred approximately 11,000 years ago (Kaptan et al., 2024). Furthermore, advanced genetic modeling, utilizing Hidden Markov with migration frameworks, estimates the divergence between domestic sheep and their wild ancestor, Asian mouflon progenitors to have occurred between 8,800 and 12,800 years ago (Li and Salehian Dehkordi, 2026a). The significant genetic heterogeneity observed across ancient and modern lineages challenges the model of a single, localized origin, instead supporting a paradigm of multiple independent domestication events derived from diverse wild stocks (Li and Salehian Dehkordi, 2026a; Li and Salehian Dehkordi, 2026b). Subsequently, centuries of anthropogenic selection based on phenotype have further driven the remarkable phenotypic diversity and high productivity characteristic of modern breeds (Thorne et al., 2021; Panigrahi et al., 2022).

Building upon this evolutionary foundation, the late 20th century was associated with the molecular genetics revolution that facilitated more targeted genetic improvement through DNA markers. The first step to establishing the linkage maps was to use the ovine genome sequence and high-density SNP arrays, which enable the transition from MAS (Marker-assisted selection) targeting major genes to genome-wide GS (Crawford et al., 1997; Ron and Weller, 2007; Legarra et al., 2015) and haplotype-based prediction for complex phenotypes (Vahedi et al., 2023). Today, sheep genomics integrates whole-genome sequencing, single-cell sequencing, transcriptomics, epigenomics, and functional genomics, profoundly advancing knowledge of economically important phenotypes such as growth, reproduction, wool quality, milk production, and disease resistance (Tan et al., 2023; Lyons et al., 2024; Ma et al., 2025; Wadood et al., 2025). Advancements in single-cell sequencing have transformed the genomics field by allowing researchers to delve into the intricate cellular heterogeneity within tissues at greater resolution (Lyons et al., 2024). This comprehensive change, with historical milestones, methodological developments, trait-specific findings, comparative ruminant genomics, and future technological directions, underscores the dynamic progress that defines current research and application in sheep breeding (Kaptan et al., 2024; Chessa et al., 2009; Hassanine et al., 2025). This review offers a comprehensive picture of the current and future state of the global sheep industry, integrating pioneering research, methodological advancements, and the latest developments in genomics. The primary objective of this narrative review is to synthesize major advances in sheep genetic mapping and functional genomics, with a specific focus on validated major genes, replicated QTL, and their biological and breeding relevance. To achieve this, the manuscript is structured as follows: Section 2 outlines the historical trajectory of sheep genomics; Section 3 details the methodological toolkit; Section 4 provides a critical, trait-specific synthesis of major genes and QTL; Section 5 discusses comparative genomics; and Section 6 explores future perspectives, including emerging technologies and economic impacts. By integrating these elements, this review aims to provide a balanced, evidence-based resource to guide future genomic improvements in sustainable sheep production.

## 2 Historical developments

Various key technological and conceptual landmarks have been used for understanding the genetic layout of sheep. The progression from coarse mapping to high-resolution genomic prediction represents the broader development of the field of genomics (Crawford et al., 1997; Sharma et al., 2015; Ma et al., 2025). A historical progression of Sheep QTL research, shown in Table 1, which clearly underscores a trend to increasing resolution, from broad chromosomal regions to single nucleotides (Ron and Weller, 2007; Legarra et al., 2015). The scale of investigation has increased from family-based studies and early marker-based diversity analyses, particularly those based on microsatellites (Esmaeil Khanian and Banabazi, 2006), to population-wide and multi-omics analyses (Tan et al., 2023; Wadood et al., 2025). Advancements at each stage have enabled breeders to more accurately predict the genetic potential of animals from their DNA.

**TABLE 1:** Historical trajectory of sheep QTL research from 1990 to 2025.

Period	Technical milestone	Representative breakthrough	Significance
1990–2000	First microsatellite linkage maps	A Texel x Coop worth resistance to Gastrointestinal parasitic nematodes breed-specific QTL was realized in a double-backcross study (Crawford et al., 1997)	Established the concept of linkage mapping in sheep, which showed that genetic markers had the ability to identify genetic areas that affected complex traits such as disease resistance
2000–2007	From QTL to quantitative trait nucleotide (QTN)	A background discussion by Ron and Weller described the strategic model of transitioning the QTL detection to the validation of the causal nucleotide QTN and the application of MAS (Ron and Weller, 2007)	Determining the exact genetic variations that cause variation in the trait, which formed the theoretical basis of more efficient breeding tools
2007–2012	First microsatellite linkage maps	A Red Maasai × Dorper sheep with a double-backcross study discovered that there were breed-specific QTL involved in resistance to the gastrointestinal parasites (Silva et al., 2012)	Supports the fact that future MAS to enhance production traits genetically will be based on the markers that are in linkage disequilibrium with these QTL
2012–2015	50K SNP arrays and population-scale GWAS	A 50K SNP array GWAS in Merino sheep identified the *NCAPG-LCORL* region on chromosome 6 as a locus of large effect on body weight, which is orthologous to height/stature loci in cattle and humans (Al-Mamun et al., 2015)	The availability of affordable, high-density SNP arrays democratized GWAS, enabling researchers to scan entire populations for associations and uncover conserved genetic mechanisms across species.
2016–2019	High-density imputation and advanced methodologies	A 3,731-Sarda ewe study used a principal component-based LD-linkage analysis (PC-LDLA) in detecting 75 QTL of milk traits, proving the power of combining pedigree and population-level linkage disequilibrium (Usai et al., 2019)	Complex statistical techniques were developed to enhance the mapping strengths and resolutions in large pedigreed populations, to improve the genetic map of complex traits such as milk production.
2020-Present	Low-coverage WGS and multi-omics integration	It is possible to combine 2,625 ovine QTL with epigenomic and chromatin conformation (Hi-C) data to prioritize causal variants using the development of resources such as the Animal GWAS Atlas (Tian et al., 2020). It has been demonstrated that lcWGS is a cost-efficient alternative to arrays to fine-map QTL, and a study of birth weight in Hu lambs has shown it to be cost-effective (Fonseca et al., 2020; Li et al., 2025; Ma et al., 2025)	Combination of several layers of biological information (multi-omics) to comprehend the role of the gene and applications of sequencing technology to grasp a greater range of genetic variation, such as rare and structural diversity

## 3 Detection methodologies: the genetic toolkit

The discovery of genes and QTL is fundamentally dependent on the available molecular and statistical tools. Development of these approaches has facilitated progress in sheep genetics (Sharma et al., 2015; Tan et al., 2023).

### 3.1 Linkage analysis

The initial effective QTL mapping technique in livestock was genetic linkage analysis, which involved the monitoring of the inheritance of genetic markers, microsatellites, and phenotypic characteristics in pedigreed families (Van Ooijen, 2004; Crawford et al., 1997). Linkage analysis represents QTL location on chromosomes by determining markers that are consistently transmitted with a phenotype. The advantages of the linkage analysis are high power to identify the QTL with large effects, the ability to withstand population stratification, and the ability to work in well-structured pedigrees. However, it has disadvantages like comparatively low mapping resolution, which usually maps QTL to wide areas of 10–20 centimorgans, involves heavy design of family structures, and ineffectiveness in the process of detecting small-effect QTL (Ron and Weller, 2007). For example, the identification of QTL of parasite resistance in Red Maasai Red x Dorper crosses, and the imaging of early scans in Spanish Churra sheep that have identified suggestive growth trait QTL with the use of microsatellite markers (Silva et al., 2012; Benavides et al., 2015; Gutiérrez-Gil et al., 2011). These studies highlight linkage analysis as a foundational tool in livestock genomics, although its limitations have driven the development of higher-resolution methods such as GWAS and GS (Legarra et al., 2015; Sharma et al., 2015).

### 3.2 Genome-wide association studies (GWAS)

With the development of high-density SNP arrays, GWAS has become the most commonly used method for QTL mapping (Sharma et al., 2015; Tan et al., 2023). GWAS scans the entire genome in populations of unrelated or distantly related individuals to identify statistical correlation between markers and traits and utilizes the advantage of the LD (Linkage disequilibrium), also known as the non-random association of alleles across loci. Its advantages include higher resolution compared to linkage analysis, it can be used to survey genetic variation in entire populations, and also to identify loci of moderate effect. However, it has disadvantages such as being susceptible to false positives because of population structure, requiring large sample sizes, and can frequently capture related genomic regions instead of the causal variants themselves, which is commonly referred to as the tag SNP problem (Ron and Weller, 2007; Sharma et al., 2015). An example use of GWAS in sheep genetics is the discovery of the cross-species conserved major growth locus identified *via* GWAS *NCAPG-LCORL* (Non-SMC Codons in I Complex Subunit G- Ligand Dependent Nuclear Receptor Corepressor Like) locus that explains body weight in Merino sheep (Al-Mamun et al., 2015; Wang et al., 2024; Majeres et al., 2024; Takasuga, 2016; Yaman et al., 2024).

### 3.3 RNA sequencing (RNA-seq)

RNA-Seq is known as a comprehensive measurement and sequencing of the entire transcriptome, which provides a dynamic snapshot of gene expression. This approach is effective in determining differentially expressed genes between animals with different phenotypes. Combined with genomic information, RNA-Seq enables the identification of functional candidate genes in large QTL regions detected by GWAS, and integrating GWAS with transcriptomic analysis provides deeper biological insight into complex traits (Zhao et al., 2024). The benefits of RNA-Seq are the ability to obtain evidence of gene participation in a functional sense, finding new transcripts and alternative splicing sites, and explaining biological pathways associated with characteristics (Bakhtiarizadeh et al., 2019; Peng et al., 2022). A notable application is re-examination of 45 publicly available RNA-Seq samples of fat- and thin-tailed sheep, to determine 103 genes under selection and to find important biological modules including angiogenesis and fatty acid oxidation, with genes such as Bone morphogenetic protein (*BMP2*) and Vascular Endothelial Growth Factor D (*VEGFD*) providing expression-supported evidence of markers related to fat deposition in the tail (Abbasabadi et al., 2024; Bakhtiarizadeh et al., 2019; Peng et al., 2022). Indeed, the PDGFD gene was also identified as a putative causal gene for tail fat deposition in sheep through transcriptome, RT-PCR, qPCR, and Western blot analyses of 248 samples of wild and domestic sheep (Li et al., 2020).

### 3.4 Whole-genome sequencing (WGS)

Whole genome sequencing (WGS) with entire genome sequencing provides the most information on genetic variation. Its advantages are an opportunity to identify variants of any type of SNPs, indels (Insertions/deletions), and larger Structural variants (SVs) such as CNVs (Copy number variations); unlike SNP arrays that aim to detect a predefined number of variants, WGS can identify all genetic variation types and provide a complete catalog of genetic variation in a population (Ma et al., 2025). Low-coverage WGS (lcWGS) imputation consider as a cost-effective strategy for large-scale studies (Casellas et al., 2021). WGS applications in sheep genomics include the identification of selective sweeps, local adaptation signatures, and functional variants affecting economically important traits. For example, high-altitude adaptation in Tibetan sheep has been associated with genes involved in cardiopulmonary and renal functions (Wei et al., 2016), while fine-mapping studies in Hu lambs identified regulatory SNPs affecting birth weight (Li et al., 2025). Whole-genome sequencing has also been used to characterize local adaptation patterns in indigenous sheep populations (Asadollahpour-Nanaei et al., 2024). These examples illustrate the accuracy and cost efficiency of WGS approaches. Despite challenges such as incomplete genome assemblies and functional annotation gaps, WGS remains an essential tool for genetic studies, livestock breeding, and conservation (Ma et al., 2025; Wang et al., 2025).

### 3.5 Microbiome-host interactions in trait expression

This interaction of the gastrointestinal microbiome on host gene expression and phenotypic trait expression in sheep, the underlying mechanism of this relationship remains to be clarified, to be regarded as a critical aspect of livestock genetics and breeding. Recently, researchers have been studying the functional interactions between the composition of microbial communities and the economically significant characteristics. An association between expression of host genes involved in energy metabolism, including *ACACA* (Acetyl-CoA carboxylase) and *FASN* (Fatty-acid synthase), and abundance of specific ruminal microbial taxa, including Prevotella and Ruminococcus has been identified that results in increased feed efficiency. Sheep with increased concentrations of these microbes showed significant positive changes in the feed ratio by 15 percent, which marks microbiome functionality in nutritional exploitation. Furthermore, the microbiome composition has a significant effect on the methane emissions, which is a key environmental and sustainability issue in ruminant production (Ferretti et al., 2026). Archaeal communities, especially Methanobrevibacter have been shown to interact with host transporters such as *MCT1* (Monocarboxylate transporter 1) and *SLC16A1* (Solute Carrier Family 16 Member 1), which mediate short-chain fatty acid transport in the rumen, resulting in a change in methane yield up to 30%. This highlights the potential effect of microbiome-guided strategies on greenhouse gas emissions. Moreover, the skin microbiome affects wool quality traits with the relative abundance of *Staphylococcus* species predicting the expression of *KRTAP*s (keratin-associated protein genes) and wool fiber diameter, and, therefore, host-microbiome interactions go beyond metabolic traits to structural phenotypes (Greeff et al., 2021).

### 3.6 Mathematical model of microbiome-host interaction

Understanding the microbiome-host axis is critical in the quantification of the interaction of the microbial communities with the host genetics in developing complex characteristics, including feed efficiency, methane emission, and resistance to diseases. This has been mathematically modeled as an extension of classical quantitative genetic models to explicitly include the effects of microbes and their interaction with host genotype and gene expression (Ferretti et al., 2026; Wadood et al., 2025). These models offer a strict level of statistical inferences and hypothesis tests on the functions of both host and microbiome in phenotype determination. Microbiome-host interaction is an example of a mathematical model that provides a quantitative framework of the interaction of genetic and microbial factors in determining complex host characteristics. The host trait, such as feed efficiency or methane emission, is expressed as a function of host genotype, microbial taxon abundance, and their interaction, formalized as:

$$y_{i}=\alpha +\beta_{1}G_{i}+\beta_{2}M_{i}+\beta_{3}\left(G_{i}\times M_{i}\right)+\varepsilon_{i}.$$

Here, the intercept ( α ) is the baseline phenotype when both genetic and microbial effects are absent, acting as a reference point for interpretation. The genotype coefficient ( $\beta_{1}$ ) is used to measure the direct effect of host hereditary variation on the trait, such as the change in feed efficiency per additional copy of a beneficial allele. The microbiome coefficient ( $\beta_{2}$ ) measures the effect of microbial abundance, for instance, the increase in methane emission associated with higher quantities of a certain archaeon in the rumen. Importantly, the interaction coefficient ( $\beta_{3}$ ) describes a phenomenon in which the effect of the host genotype on the phenotype depends on microbial presence, and *vice versa*. For example, a genotype may decrease methane production only in the presence of a specific microbial community, revealing synergistic or antagonistic dynamics. The error term ( $\varepsilon_{i}$ ) represents the residual biological and environmental variation that is not explained by the measured variables of the host and micro-environment, which allows statistical inference to determine the proportion of phenotypic variance each component contributes. Statistical R code implementations with simulated data illustrate these concepts, enabling researchers to test hypotheses regarding microbiome-host interactions and to estimate their effects rigorously. Ultimately, each coefficient in the model reflects a distinct biological mechanism, $\beta_{1}$ and $\beta_{2}$ for the main genetic or microbial effects, $\beta_{3}$ for emergent host-microbe dependencies, allowing for a nuanced interpretation of how molecular, ecological, and evolutionary forces combine to determine trait variation in animals and other organisms. The following is a linear model of the expression level of a host gene or measured phenotype y:

$$y_{i}=\alpha +\beta_{1}G_{i}+\beta_{2}M_{i}+\beta_{3}E_{i}+\beta_{4}\left(G_{i}\times M_{i}\right)+\varepsilon_{i}$$

**FIGURE 1:**
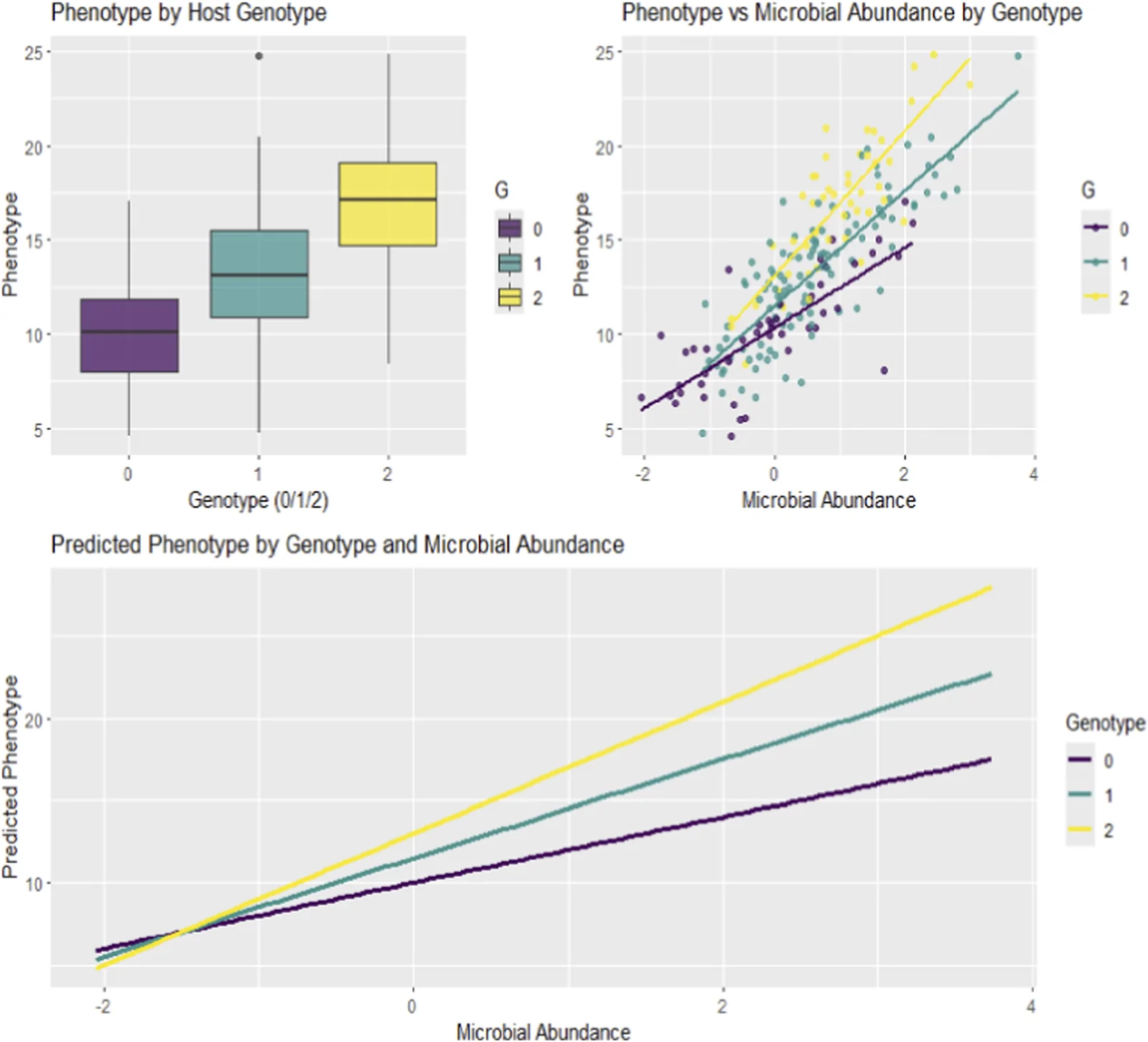
Conceptual visualization of the fixed-effect linear model for host-microbiome-QTL interactions. The data and parameters depicted are based on a conceptual synthesis of published sheep genomic pathways and simulated interactions designed to illustrate the theoretical framework, rather than representing a single empirical dataset.

Where: $y_{i}$ = Expression level of a host gene or measured phenotype for individual i, $G_{i}$ = Host genotype at a specific locus (coded, e.g., as 0/1/2), $M_{i}$ = Abundance (relative or absolute) of a microbial taxon (e.g., *via* 16S rRNA read counts or log-transformed abundances), $E_{i}$ = Environmental or covariate effects (optional, can be omitted for a minimal model), α = Intercept, $\beta_{1}$ , $\beta_{2}$ , $\beta_{3}$ = Main effect coefficients, $\beta_{4}$ = Interaction coefficient capturing G × M (genotype-microbe) effects, $\varepsilon_{i}$ = Residual error (assumed to be normally distributed). This model is able to estimate the role of microbiome-host interaction in phenotypic variation in phenotypes such as feed efficiency and methane emissions (Figure 1). While the fixed-effect model in Figure 1 estimates specific directional effects ( β coefficients), translating discoveries into GS and marker MAS requires partitioning the phenotypic variance into random components to estimate heritability and predict breeding values. Therefore, we extend this framework to advanced variance components. The total phenotypic variance ( $\sigma_{P}^{2}$ ) is partitioned as: $\sigma_{P}^{2}=\sigma_{g}^{2}+\sigma_{m}^{2}+\sigma_{g\times m}^{2}+\sigma_{e}^{2}$ . To capture the complex ecological dynamics of the rumen and gut in sheep, the microbiome variance ( $\sigma_{m}^{2}$ ) can be further decomposed into taxonomic variance ( $\sigma_{m,tax}^{2}$ ), for example, derived from 16S rRNA profiles, and functional variance ( $\sigma_{m,func}^{2}$ ), or derived from microbial metabolic pathways (e.g., volatile fatty acid production). Furthermore, to account for environmental adaptation—a critical factor in sheep production—this model incorporates a microbiome-by-environment interaction ( $\sigma_{m\times e}^{2}$ ), yielding a comprehensive, multi-layered variance structure: $\sigma_{P}^{2}=\sigma_{g}^{2}+\sigma_{m,tax}^{2}+\sigma_{m,func}^{2}+\sigma_{g\times m}^{2}+\sigma_{m\times e}^{2}+\sigma_{e}^{2}$ . In this advanced framework, the sum of $\sigma_{m,tax}^{2}$ and $\sigma_{m,func}^{2}$ defines the microbiability ( $m^{2}=\sigma_{m}^{2}/\sigma_{P}^{2}$ ), representing the proportion of phenotypic variance explained by the microbiome. The term $\sigma_{g\times m}^{2}$ quantifies the inter actionability, reflecting the extent to which host QTL effects are contingent upon specific microbial profiles. Integrating these advanced variance components into genomic prediction is not merely a statistical exercise; it is essential for the accurate selection of low-heritability traits like disease resistance. If $\sigma_{g\times m}^{2}$ is substantial, standard genomic best linear unbiased prediction (GBLUP) models that ignore the microbiome will systematically misestimate the breeding values of host QTL. By utilizing Microbiome-Adjusted Genomic BLUP (M-GS) models, breeders can capture the hidden heritable variance residing in the host-microbiome whole genome. This mathematical integration ensures that the deployment of major genes and QTL is optimized, directly linking molecular discoveries in the microbiome-host axis to measurable, economically viable outcomes in modern sheep breeding programs.

### 3.7 Structural variants and complex genomic rearrangements

The genetic variation on the microbiome population in relation to SVs, including CNVs, inversions, and translocations, contributes to phenotypic variation among sheep. The SVs are regulatory functions in economically important traits. Moreover, chromosomal inversions have been associated with key reproductive adaptations. For example, there is a four mega base inversion on chromosome 10, including the *MTNR1A* (Melatonin Receptor 1A) gene, which regulates melatonin signaling pathways, in high-latitude sheep breeds but is absent in equatorial populations, implicating a role in seasonal breeding patterns adapted to photoperiod variations (Marcos-Carcavilla et al., 2008; Marcos-Carcavilla et al., 2010). It is also shown that immune traits can be affected by structural rearrangements like reciprocal translocations. A translocation involving chromosomes 5 and 12 of Red Maasai sheep disrupts the IL2 gene, which is a key mediator of immunological responses, and correlates with enhanced resistance to gastrointestinal parasitic infections (Benavides et al., 2015). These findings underscore the importance of integrating SV analysis into sheep genomics research to fully capture the genetic architecture underlying complex traits and inform breeding strategies aimed at improving production, reproduction, and disease resilience.

### 3.8 Emerging approaches and integrated methodologies

The future of sheep genetics is the use of emerging technologies that are based on the combination of various approaches. Advanced GWAS methods, such as PC-LDLA (Principal component-based LD-Linkage analysis), which combines linkage and linkage disequilibrium analysis, resulting in increased mapping resolution in complex pedigrees, have been approved on milk characters in Sarda ewes (Usai et al., 2019). BayesR and other multi-trait GWAS models can simultaneously analyze multiple traits to identify pleiotropic QTL, evidenced by 206 pleiotropic wool trait QTLs in Merino sheep (Sheep Genetics Annual Outcomes Report, 2023–24). Integration of multi-omics and functional genomics is essential in focusing on causal variants; the Animal GWAS Atlas is an example of this combination of ovine QTL with chromatin accessibility (ATAC-seq) (Yan et al., 2024), histone modifications (ChIP-seq) (Chen et al., 2024) and 3D genome architecture (Hi-C) (Xu et al., 2025)to visualize functional non-coding variants(Fonseca et al., 2020). Cross-species epigenomics, including DNA methylation atlases for cattle, sheep, and goats, further elucidates conserved regulatory elements (Wadood et al., 2025). Gene editing *via* CRISPR-Cas9 (Clustered regularly interspaced short palindromic repeats) provides functional validation through making a precise knockout of the gene of interest. Recent developments have seen the establishment of a long-wool/double-muscle sheep using *MSTN* (Myostatin) and *FGF5* (Fibroblast growth factor 5) knockouts. Advances in making the editing process more efficient and less mosaic are making gene editing feasible in the livestock breeding field (Crispo et al., 2015; Kalds et al., 2022). A comprehensive summary of the major structural variants, genomic regions, epigenetic modifications, and economic impacts discussed across traits is provided in Tables 2-7.

**TABLE 2:** Major structural variants in sheep and their effects.

Trait category	Chromosome/genomic region	Gene/locus	Study type	Breed/population	Sample size	Reported effect	Validation status	References
Milk production	Chr14: 24.5–25.1 Mb	DGAT1	CNV Analysis	Chinese goats^a^	NR	Duplication increases fat by 0.3%	Cross-species inference	Liu et al. (2023)
Reproduction	Chr10: 15.2–19.2 Mb	MTNR1A	WGS/Array	Temperate breeds	NR	Fixed in temperate breeds	Replicated QTL	Marcos-Carcavilla et al. (2008)
Disease resistance	t(5; 12)	IL2	Linkage	Red Maasai	NR	Enhanced parasite resistance	Candidate association	Benavides et al. (2015)
Wool quality	Chromosome 3	KRTAP	WGS/Array	NR	NR	Affects fiber diameter and strength	Candidate association	Bai et al., 2024; Sun et al., 2024
Growth	Chromosome 6	NCAPG-LCORL	GWAS	NR	NR	Influences body size and muscle	Replicated QTL	Takasuga, 2016; Majeres et al., 2024

^a^ The DGAT1 CNV reference pertains to goats; included here as cross-species inference.

NR, Not Reported in primary literature; NR, not reported.

**TABLE 3:** Genomic regions associated with climate resilience traits.

Trait category	Chromosome/genomic region	Gene/locus	Study type	Breed/population	Sample size	Reported effect	Validation status	References
Heat tolerance	OAR3	HSP90AA1	GWAS	Australian Merino	NR	Reduces rectal temp by 0.5 °C	Candidate Association	Marcos-Carcavilla et al. (2010)
Drought resistance	OAR6	LEPR	Selective Sweep	Fat-tailed Breeds	NR	20% better weight maintenance	Candidate Association	Deniskova et al. (2019)
Parasite resistance	OAR20	MHC-II	GWAS/Selection Sig.	Variable Rainfall Regions	NR	Reduces fecal egg count by 40%	Replicated QTL	Villegas-Castañeda et al. (2024)
High-altitude adapt.	OAR2	EPAS1	WGS	Tibetan Sheep	NR	Enhances oxygen metabolism	Replicated QTL	Wei et al. (2016)
Cold adaptation	Multiple (OAR1, 10)	UCP, RXFP2	Selective Sweep	Mediterranean/High-Alt.	NR	Improves thermoregulation	Candidate Association	Ahmad et al., 2021; Tsartsianidou et al., 2021

*NR, Not Reported EPAS1 (*Endothelial PAS, domain-containing protein 1*)*.

**TABLE 4:** Summary of key genes and QTL for economic traits in sheep.

Trait category	Chromosome/Genomic region	Gene/Locus	Study type	Breed/Population	Sample size	Reported effect	Validation status	References
Growth	OAR6	*NCAPG-LCORL*	GWAS	Australian Merino	NR	Major locus; ∼2 kg effect	Replicated QTL	Al-Mamun et al. (2015)
Growth	OAR16	*GHR*	Linkage	Spanish Churra	NR	Suggestive QTL for stature	Candidate Association	Gutiérrez-Gil et al. (2011)
Reproduction	OAR6	*BMPR1B*	WGS/CRISPR	Booroola/Hu	NR	Increased ovulation/litter size	Experimentally Validated	Zhou et al., 2018; Zhang et al., 2020
Reproduction	Multiple	*PI3K-Akt, SMAD*	Meta-GWAS	Multiple	NR	Ovarian follicle pathways enriched	Candidate Association	Ghiasi and Abdollahi-Arpanahi (2021)
Reproduction	Multiple	*SH2B1*	GWAS	Merino Rams	NR	Candidate for semen quality	Candidate Association	Hodge et al. (2023)
Wool quality	Multiple	*KRTAP, FGF5*	Multi-trait GWAS/CRISPR	Merino	NR	Major loci for fiber diameter/length	Replicated/Validated	Bai et al., 2024; Kalds et al., 2022
Milk production	OAR3	*LALBA*	LDLA/Linkage	Multiple	NR	Causal variant for protein %	Experimentally Validated	Zhou and Dong, 2013; Usai et al., 2019
Milk production	OAR3	*SOCS2*	GWAS	French Dairy	NR	Antagonistic pleiotropy	Replicated QTL	Rupp et al. (2015)
Disease resistance	OAR20	*PRNP*	Sequencing/Linkage	Multiple	NR	Primary determinant of scrapie	Experimentally Validated	Bishop and Morris (2007)
Disease resistance	OAR2, 3, 6	Multiple (Innate Immunity)	Linkage/GWAS	Red Maasai/Djallonké	NR	GIN resistance/anemia variance	Candidate Association	Benavides et al., 2015; Alvarez et al., 2019

NR, Not Reported; LDLA, Linkage-LD, Analysis; GIN, gastrointestinal nematodes.

**TABLE 5:** Epigenetic modifications associated with key traits in sheep.

Trait category	Chromosome/genomic region	Gene/locus	Study type	Breed/population	Sample size	Reported effect	Validation status	References
Muscle growth	OAR2	MSTN promoter	DNA Methylation	Texel	NR	Hypermethylation reduces expression	Candidate Association	Qiu et al., 2024; Clop et al., 2006
Wool quality	Multiple	KRTAP cluster	Histone Mod. (H3K27ac)	NR	NR	Enhances keratin gene expression	Candidate Association	Wadood et al. (2025)
Fat deposition	Multiple	ABCA1	miRNA (miR-33a)	NR	NR	Regulates lipid metabolism	Candidate Association	Bakhtiarizadeh et al. (2019)
Reproduction	OAR6	BMPR1B	DNA Methylation	NR	NR	Methylation affects ovulation rate	Candidate Association	Zhang et al. (2020)
Milk production	Multiple	Casein Cluster	Histone Modifications	NR	NR	Influences yield via regulatory elements	Candidate Association	Wadood et al. (2025)

NR, not reported.

**TABLE 6:** Functional validation studies for major sheep genes.

Trait category	Chromosome/genomic region	Gene/locus	Study type	Breed/Population	Sample size	Reported effect	Validation status	References
Muscle growth	OAR2	MSTN	CRISPR-Cas9 KO	NR	NR	20% increase in muscle mass	Experimentally Validated	Crispo et al., 2015; Kalds et al., 2022
Wool length	OAR11	FGF5	CRISPR-Cas9 KO	NR	NR	30% longer wool fibers	Experimentally Validated	Kalds et al. (2022)
Reproduction	OAR6	BMPR1B	Transgenic Overexp.	NR	NR	Increased ovulation rate	Experimentally Validated	Zhou et al., 2018; Zhang et al., 2025
Disease resistance	OAR20	PRNP	RNAi Knockdown	Bovine*	NR	Reduced prion accumulation	Cross-Species Inference	Sutou et al. (2007)
Disease resistance	Multiple	SP110	Transgenic Overexp.	NR	NR	Enhanced Salmonella resistance	Experimentally Validated	Bishop and Morris (2007)

*The PRNP RNAi, study was conducted in bovine models; included as cross-species functional inference. NR, not reported.

**TABLE 7:** Economic benefits of genomic selection in sheep breeding programs.

Program/Region	Trait	Genetic gain increase	ROI/Genetic gain	References
New Zealand (SIL)	Growth	35%	280%	Sheep Improvement Limited, 2020
New Zealand (SIL)	Meat	30% reduction in generation interval; 25% genetic gain	250% ROI over 10 years	Sheep Improvement Limited, 2020
Australia (Merino)	Wool quality	25%	220%	Wicki et al. (2024)
US Sheep GEMS	​	>0.60 prediction accuracy for resilience	Feed-cost savings (673,651 t/year)	USDA NIFA, 2022
French Dairy Sheep	​	Improved mastitis resistance	40% increase in genetic gain	Oget et al. (2019)
UK (Texel)	Muscle mass	40%	300%	British Texel Sheep Society, 2018
China (Hu sheep)	Reproduction	30%	190%	Li et al. (2024)

## 4 Trait-specific genes and QTL: dissecting the ovine phenome

The application of the methodologies described above has led to the discovery of a multitude of genes and QTL influencing key economic traits. This section provides a detailed review of these findings, organized by trait category.

### 4.1 Environmental adaptation genomics

The environment induced considerable challenges to sustainable sheep production. Exposure to environmental stressors threatens animal health, productivity, and welfare, in turn highlighting the importance of identifying and breeding for resilience traits. Emerging evidence has started to explain the genetic basis supporting such adaptive traits, providing factors for selection to improve climate resilience. An example is a GWAS study that identified a QTL on chromosome 3, including the *HSP90AA1* gene in Australian Merino sheep, explaining around 12% of the phenotypic variation in rectal temperature during heat stress situations. Interestingly, the favorable allele of the locus is found at higher frequencies in equatorial breeds, reflecting local adaptation to warm environments (Marcos-Carcavilla et al., 2010). In the context of drought resilience, studies in fat-tailed sheep breeds have shown a selective sweep on chromosome six harboring the leptin receptor gene (*LEPR*), which plays an essential role in fat metabolism and energy mobilization during periods of feed scarcity (Deniskova et al., 2019). The homozygous sheep with the beneficial allele respond to drought stress with a 20 percent better weight gain, which indicates the practical importance of this genotype. Moreover, the resistance of parasites towards the evolving climatic patterns has been associated with genomic regions with immune-related genes like the major histocompatibility complex (MHC) class II, where the presence of selection signatures has been observed in breeds of the region where the rainfall variability is increasing (Villegas-Castañeda et al., 2024). These results underscore the possibility of using genomic knowledge to increase the soundness of sheep populations to the multiple pressures of climate change and demonstrate how breed-specific genomic resources can support local adaptation programs (Asadollahpour-Nanaei et al., 2024), which can be used to develop breeding schemes that enhance animal resilience and productive sustainability. In addition to heat and drought, analysis of genomes has found that there are conserved regions among Mediterranean sheep breeds that include ones on OAR1 and OAR10, which are related to local adaptation to arid conditions. These areas contain such genes as *RXFP2*, which control the morphology of horns and thermoregulation, which is why breed-specific selection is crucial to overcome climate change (Tsartsianidou et al., 2021).

The evidence for climate resilience traits is currently dominated by selective sweep analyses and candidate gene associations (e.g., *HSP90AA1*, *LEPR*) in specific indigenous breeds. While the biological plausibility is high, the reproducibility of these QTL across commercial breeds remains unverified. The practical breeding value is currently limited to localized conservation and crossbreeding programs aimed at introgressing adaptive alleles into commercial flocks, rather than widespread GS. Several studies used reported candidate SNPs under climate-mediated selection, which were significantly correlated with climatic variables across a broader global set of native populations (LV et al., 2014; Cao et al., 2020; Salehian-Dehkordi et al., 2023). The candidate genes often are linked to signals involved in energy metabolism and endocrine or autoimmune regulation, higher genomic diversity, climate adaptation, and resistance to pneumonia (LV et al., 2014; Cao et al., 2021). Moreover, the global frequencies of the core *TBC1D12* haplotype (*TBC1D12-CH1*) and the OAR22_18929579-A allele displayed significant geographic patterning and climate-driven correlations (LV et al., 2014). Using large-scale SNP and whole-genome data from domestic sheep and wild Ovis relatives, clear evidence of gene flow found from wild species into local domestic populations (Cao et al., 2020). The introgressed alleles often involved genes related to smell and immunity, especially *ADCY3, TRPV1*, and the *PADI* gene family, with *PADI2* highlighted as a key candidate (Cao et al., 2020). The selection signatures suggested that this region also underwent positive selection in resistant populations (Cao et al., 2020). Overall, the introgression from wild relatives was not just a historical event, but a functional force that shaped adaptive traits in domestic sheep, especially immunity and environmental tolerance (Cao et al., 2020).

Salehian-Dehkordi added a structural-variation perspective by mapping 39,145 CNVs in 47 old autochthonous populations genotyped with high-density 600K SNP data (Salehian-Dehkordi et al., 2023). It identified 136 deletions and 52 duplications associated with climatic variables, with notable links to solar radiation and candidate genes related to heat stress, cold adaptation, wool traits, DNA repair, metabolism, reproduction, growth, and immune response. The same study also found enriched pathways related to nucleotide, protein-complex, and GTPase activity, and overlap between climate-associated CNVs and 140 known sheep QTLs, supporting their value as potential markers for climate-adapted breeding (Salehian-Dehkordi et al., 2023).

### 4.2 Growth, body weight, and carcass traits

Growth rate and body size are the main traits influencing the efficiency of the meat-sheep flock that is directed by a highly polygenic architecture, but with several major loci that have been identified. The *NCAPG-LCORL* locus on chromosome 6 (OAR6) was the most significant and was replicated in sheep as a QTL of body size. A landmark GWAS in Australian Merinos revealed a strong association in this region, where the lead SNP accounted for about 0.25 standard deviations of mature body weight, corresponding to approximately a 2 kg effect (Al-Mamun et al., 2015). The locus contains the *NCAPG* and *LCORL* (Ligand Dependent Nuclear Receptor Corepressor Like) genes, which are regulators of stature and body size and are preserved in all the species, such as cattle, horses, and humans (Takasuga, 2016; Majeres et al., 2024). The mTOR-IGF signaling pathway is considered a biological mechanism involved in fetal myogenesis and postnatal muscle development. Other significant loci in body weight and muscle growth have been identified in chromosome 2, chromosome 3, chromosome 6, and chromosome 18, with the identification of the *ADGRB3, CAPN2*, and *RELN* genes in selective sweeps between domestic and wild sheep (Asadollahpour-Nanaei et al., 2024). Spanish Churra sheep linkage scan revealed QTL of stature in OAR6, OAR16, and OAR23, and a Growth Hormone Receptor (GHRI) gene is a candidate near the OAR16 QTL (Gutiérrez-Gil et al., 2011). A recent low-coverage whole-genome sequencing study in Hu lambs fine-mapped a regulatory SNP near PLAG1 significantly influencing birth weight (+0.33 kg), demonstrating the power of sequence-level resolution for complex trait dissection (Li et al., 2025). The *NCAPG-LCORL* locus represents the gold standard for evidence strength and reproducibility in ovine growth traits, consistently replicated across multiple breeds and species. However, the mapping resolution for other growth QTL remains relatively broad. The practical breeding value for growth is exceptionally high, not through MAS for individual QTL, but because these highly heritable, well-mapped loci significantly boost the accuracy of whole-genome GS prediction models in commercial meat sheep.

### 4.3 Reproduction and fertility

Reproductive efficiency traits, including fertility, prolificacy, and semen quality, are important in flock productivity but have low heritability, which makes genomic tools essential. Prolificacy genes have been identified in Booroola Merino and Hu sheep; a meta-GWAS on litter size revealed 57 loci that were enriched with ovarian follicle development pathways genes, such as the *PI3K-Akt* and *TGF-β* signaling (SMAD2/3) (Ghiasi and Abdollahi-Arpanahi, 2021). Chinese breeds WGS identified missense variants in *BMPR1B* (Bone morphogenetic protein receptor type 1B), *CC2D1B*, and *CCDC63* correlated with litter size variation (Zhong et al., 2024). Merino rams’ studies introduced 35 QTL for semen traits linked to neuroendocrine genes like *SH2B1*, which regulate leptin and insulin pathways involved in spermatogenesis (Hodge et al., 2023). The genes such as *PAK1*, *CYP19A1,* and *PER1* are seasonal reproduction adaptations, and SNPs with breed-specific variations in Northwestern Chinese sheep were found (Zhu et al., 2023). These discoveries offer encouraging genomic indicators to move forward on the selection of enhanced reproductive characteristics, even with their complexity. Reproductive traits present a stark contrast in evidence strength. The *BMPR1B* mutation is a fully validated, causal major gene with immense practical breeding value *via* MAS. Conversely, the numerous QTL associated with semen quality and general litter size identified *via* GWAS suffer from low mapping resolution and low heritability. The practical breeding value for these polygenic reproductive traits relies entirely on multi-trait GS models, as individual candidate markers currently lack the robustness required for commercial MAS. Recent whole-genome sequencing of 297 Duolang sheep has further expanded genomic resources for identifying variants associated with litter size and provides valuable data for improving reproductive traits in sheep (Fang et al., 2025).

### 4.4 Wool and fleece quality

Most sheep breeds are characterized by wool quality, and such characteristics as fiber diameter, staple strength, and fleece weight have a key impact on the value of the products. Multi-trait GWAS in Australian Merinos found 206 pleiotropic QTL that influence various wool traits, including the key candidate genes of the *KRTAP* clusters, which are crucial structural components of wool fibers and the regulatory ones, such as *FGF5* and *STAT3* (Sheep Genetics Annual Outcomes Report, 2023–24). Earlier targeted linkage scans in Merino families detected QTL for fiber diameter and staple strength on chromosomes 3, 4, 11, and 25 near *KRTAP* gene clusters (Bidinost et al., 2008; Phuaa et al., 2015). CRISPR-Cas9 knockouts of *FGF5* alongside *MSTN* have produced sheep with a stable long-wool phenotype, demonstrating the potential of gene editing to rapidly introduce desirable fleece traits (Kalds et al., 2022). Moreover, selective sweep analyses in high-altitude breeds like the Changthangi sheep reveal co-selection of genes influencing fleece properties and thermoregulation, such as *UCP2/3* (uncoupling proteins) involved in mitochondrial thermogenesis) and *COL1A1* (collagen), suggesting genetic adaptation for both insulation and metabolism in cold, hypoxic environments (Ahmad et al., 2021; Bai et al., 2024; Sun et al., 2024). Recently, new sheep reference genome (T2T-sheep1.0) assembly corrected several structural errors in previous reference assemblies and identified new SNP at the end of chromosome 20, which improved the population genetic analyses and detection of selective signals for wool fineness (*FOXQ1* gene) by supporting the π ratio and allele frequencies between fine-wool and hairy sheep (Luo et al., 2025). They applied both SNPs and structural variants to detect selection signatures among hairy and coarse-wool, medium-wool and fine-wool domestic sheep populations with decreasing fleece fibre diameters, which lead to identifying 228 shared selected genes, including *TP63, KRT* (*KRT77, KRT1, KRT2, KRT74* and *KRT71*) and *IRF2BP2* (Luo et al., 2025).

The genetic architecture of wool quality is highly polygenic, with evidence strength heavily concentrated in structural gene clusters (e.g., *KRTAPs*). While functional validation (e.g., *FGF5* CRISPR knockouts) has proven the biological mechanism of fiber length, the reproducibility of specific GWAS-identified SNPs across different Merino sub-populations is variable. The practical breeding value is currently realized through high-density GS panels that capture the cumulative effect of these fiber-structure loci, rather than through individual marker diagnostics.

### 4.5 Milk production and composition

Milk yield and composition, including protein and fat content, are the main selection objectives of profitability in dairy sheep breeds. Linkage studies in East Friesian × Dorset backcrosses introduced QTL for milk yield on chromosomes 2, 12, 18, and 20, which have loci on OAR2 and OAR20 (Zhang et al., 1998). A high-resolution analysis in Sarda ewes using PC-LDLA identified 75 milk-related QTL, though each accounted for less than 3% of phenotypic variance, highlighting the polygenic nature of milk production (Usai et al., 2019). The first significant discovery was the identification of a causal SNP in the *LALBA* gene that influences milk protein percentage and was confirmed by combined LDLA and linkage mapping, which was a specific marker of MAS (Zhou and Dong, 2013; Banos et al., 2019). Additionally, a variant in the suppressor of cytokine signaling-2 (*SOCS2*) gene on OAR3 exemplifies genetic pleiotropy, with one allele boosting the number of somatic cells (a mastitis marker) and another allele positively affecting growth, an example showing antagonistic swamps between growth hormone and cytokine signaling pathways (Rupp et al., 2015).

Milk production genomics reveals a highly polygenic architecture with limited evidence for large-effect replicated QTL, aside from the *LALBA* (Alpha-lactalbumin) causal variant and the pleiotropic *SOCS2* region. The mapping resolution for milk yield remains a challenge due to the complex regulatory networks involved. Consequently, the practical breeding value of individual milk QTL is low; instead, the industry relies on GS to capture the infinitesimal effects of thousands of loci, highlighting a gap between biological discovery and direct MAS application.

### 4.6 Disease resistance

One of the strategies to reduce economic losses in sheep production is breeding against diseases to enhance the welfare of animals. Preserving genetic diversity within livestock populations maintains a capacity to respond to changing environments and rapidly evolving pathogens (Vasoya et al., 2024). One of the strategies to reduce economic losses in sheep production is breeding against diseases to enhance the welfare of animals. The Ovine Major Histocompatibility Complex, abbreviated as Ovar-MHC is an important source of immunogenetic diversity, and consists of a highly polymorphic set of genes that play a critical role in adaptive or innate immune responses and is located on the long arm of chromosome 20 in sheep (Vasoya et al., 2024). Based on functional structure, the Ovar-MHC is categorized into three primary classes. In ovine studies, the primary focus remains on MHC Class II, which is the most significant and extensively studied region in ovine immunogenetics, as polymorphisms in these genes, particularly *DRB1*, are directly linked to resistance against parasites and infectious diseases and have significant impact on host disease resistance (Huang et al., 2022; Salim et al., 2024). Ovar-DRB is the most widely studied gene in sheep, and polymorphisms in exon 2 of this gene (which encodes the antigen-binding region) determine the ability of the immune system to recognize various pathogens (Özdil et al., 2018; Salim et al., 2024). Several studies on breeding for resistance to gastrointestinal parasitism in sheep have led to the identification of various single nucleotide polymorphisms (SNPs). Potentially, genes in close proximity to the Major Histocompatibility Complex (MHC), such as those encoding interferons (e.g., *IFN-γ*) and interleukins (e.g., *IL-4, IL-10*, and *IL-17*), are key components of the immune system (Huang et al., 2022; Pacheco et al., 2024). Given the pivotal role of the MHC in regulating immune responses, these genes may play a specific role in modulating resistance to parasitic infections.

The genetic control of disease in sheep in scrapie resistance is well established, and mostly relies on polymorphisms at codons 136, 154, and 171 of the *PRNP* (Prion Protein) on chromosome 20 (OAR20) (Bishop and Morris, 2007). For instance, QTLs near the heat shock protein gene *HSP90AA1* on OAR17 will adjust disease incubation periods (Marcos-Carcavilla et al., 2008). Resistance to gastrointestinal nematodes (GIN) is essential for productivity, especially in tropical pastures. A genetic analysis of Red Maasai × Dorper sheep showed that six QTL possessed all the beneficial alleles that are found in the resilient Red Maasai breed (Benavides et al., 2015). Recently, 22 SNPs explaining up to 34% of the variance in anemia were discovered in Djallonké lambs in which the innate immunity gene *MBL2* and tissue repair gene *ADAMTS17* were involved (Alvarez et al., 2019). The other obstacle in sheep breeding is mastitis resistance. GWAS in French dairy sheep determined key QTL on OAR3 (*SOCS2*), OAR2, OAR16, and OAR19 that influence the somatic cell count (SCC), with antagonistic effects observed between SCC and milk yield at certain loci, which requires balanced selection methods (Oget et al., 2019). These developments show how genetics has been incorporated in breeding activities to boost resistance against major sheep diseases. Even though GS has very significant advantages, issues of smallholder systems remain a challenge because of the cost of genotyping. International consortia, such as the International Sheep Genomics Consortium, have mitigated this by pooling data, achieving prediction accuracies of 0.2–0.3 for low-heritability traits like fertility. In South African Merino, GS has led to a 1.5-fold increase in production traits, underscoring its role in sustainable breeding (Nel et al., 2024).

Disease resistance genomics is bifurcated. The *PRNP* polymorphisms for scrapie resistance represent the highest level of evidence, reproducibility, and practical MAS application in sheep. However, for complex diseases like gastrointestinal nematodes (GIN) and mastitis, the evidence consists largely of population-specific candidate QTL (e.g., *MHC* regions, *MBL2*) with low mapping resolution and high genotype-by-environment interactions. The practical breeding value for these complex diseases currently depends on expanding multi-breed reference populations to improve GS accuracy, as single-marker MAS is not yet viable.

### 4.7 Epigenetic regulation of economic traits

Epigenetic modifications are key regulators of genotype-environment interactions and regulate the expression of genes that are important in economically significant traits in sheep (Wang et al., 2024). DNA methylation is one of the most important epigenetic processes that has been shown to affect the growth of muscle by changing the level of expression of genes. For instance, *MSTN* gene promoter differentially methylated sites are associated with reduced *MSTN* expression and subsequent muscle mass gain in Texel sheep (Clop et al., 2006), providing compelling epigenetic support of the callipyge phenotype. Additionally, histone modifications such as histone H3 lysine 27 acetylation (*H3K27ac*) are also reported to be the indicators of active enhancers in the *IGF2* gene locus, which is an essential gene that regulates growth (Wadood et al., 2025). The *H3K27ac* breed-specific enrichment in sheep muscle tissue identified through ChIP-seq has shown that H3K27ac is capable of growth QTL regulation and is part of the phenotypic variation (Wadood et al., 2025). Furthermore, non-coding RNAs, particularly microRNAs (miRNAs), play a significant role in the post-transcriptional regulation of muscle development. It has been shown that miR-1 and miR-133 regulate genes involved in the myogenesis pathway, and miR-206 is directly specific to the 3′untranslated region (UTR) of *MSTN*, thereby fine-tuning its expression (Ghafouri et al., 2024; Qiu et al., 2024). GWAS of complex traits and diseases in livestock have identified several genetic variants, the majority of which are located in non-coding genomic regions. Although the involvement of numerous regulatory elements (REs) in various tissues of domestic animals has been predicted, their evolutionary conservation and effects on complex traits (especially in ruminants) have not been fully elucidated (Chen et al., 2024). To address this gap, Chen et al. (2024) analysed epigenomic and transcriptional datasets from the livers of six mammals to identify regulatory sequences. The integration of these sequences with multi-omics datasets, such as gene expression, chromatin accessibility, and DNA methylation, revealed that REs with varying degrees of evolutionary conservation across species show distinct enrichments for GWAS signals associated with complex traits.

These dynamic processes of gene expression are controlled by epigenetics, and they, in turn, determine major characteristics of the organism, including muscle development in sheep, and highlight the importance of incorporating epigenetic knowledge to complement current knowledge of genetic pathways of economically important traits.

### 4.8 Mathematical modeling of gene networks

Systems biology approaches have shown that economically valuable phenotypes in sheep are regulated by complex gene networks rather than individual gene effects, highlighting the complexity of genetic regulation of phenotypic expression (Ghafouri et al., 2024). These networks can be mathematically modeled to offer an effective method of explaining the dynamic interactions of multiple QTL and regulatory genes, and hence offer mechanistic explanations of how traits develop. It has been shown that the growth trait network based on the mTOR signaling pathway integrates the signals of key QTL such as *NCAPG-LCORL*, *IGF1*, and *MSTN*, which regulate muscle development (Takasuga, 2016; Qiu et al., 2024). A system of ODEs (Ordinary differential equations) can be used to describe this network in a quantitative manner to reflect the temporal dynamics of protein concentrations and interactions. The model simulations suggest that an increase in *IGF1* expression by 20% leads to a muscle mass increase of approximately 15%, which is close to observed empirical data. In the same way, wool quality is regulated by a hierarchical gene regulatory network involving *KRTAPs* and their upstream modulators like *FGF5* and *SOX9*. The model of the Boolean networks provides *FGF5* as a master regulator, and its inhibition is predicted to maintain long-term wool growth, providing genetic or biotechnological intervention targets (Bai et al., 2024; Sun et al., 2024). The ovulation rate and other reproductive characteristics are regulated by a complex regulatory cascade in the BMP/TGF-2 signaling pathway, including *BMP15*, *GDF9* (Growth differentiation factor 9), and *BMPR1B*. Bayesian network analyses underscore *BMPR1B* principal role in litter size variability (Ghiasi and Abdollahi-Arpanahi, 2021; Zhang et al., 2020). These mathematical and computational models bridge the gap between genetic markers and phenotypic mechanisms by revealing how multiple loci interact dynamically to shape economic strategies (Ghafouri et al., 2024). The mTOR signaling pathway is a combination of the signals of various QTLs, such as *NCAPG-LCORL, IGF1*, and *MSTN*, which participate in controlling muscle development. This network can be modeled by a system of ODEs:

$$\left\{\frac{d\left[mTOR\right]}{dt}=k_{1}\left[IGF1\right]-k_{2}\left[mTOR\right]\frac{d\left[p70S6K\right]}{dt}=k_{3}\left[mTOR\right]-k_{4}\left[p70S6K\right]\frac{d\left[MSTN\right]}{dt}=k_{5}-k_{6}\left[MSTN\right]-k_{7}\left[mTOR\right]\left[MSTN\right]\right.$$

Where $k_{1}$ - $k_{7}$ are rate constants, and brackets denote concentrations. This model predicts that a 20% increase in IGF1 expression leads to a 15% increase in muscle mass, consistent with empirical observations.

### 4.9 Functional genomics validation approaches

To ensure causal relationships between genetic variants and observed phenotypic characteristics, functional validation is a crucial step that would take the field of associative studies beyond and develop the mechanistic knowledge in sheep genomics. Various approaches have been efficiently applied in sheep to check the functionality of the genes and how they affect economically significant traits. Interestingly, CRISPR-Cas9 genome editing has been applied to produce targeted knockouts, including *MSTN* gene disruption, which caused a severe phenotype of spontaneous, 20% increase in muscle mass with decreased fat deposition, which is a confirmation of inhibitory activity of *MSTN* in muscle development (Crispo et al., 2015; Kalds et al., 2022). Similarly, inhibition of the *FGF5* gene *via* CRISPR produced sheep exhibiting approximately 30% longer wool fibers, validating its function in hair growth regulation (Kalds et al., 2022). Moreover, the presence of RNA interference (RNAi) strategies based on the siRNA-induced suppression of *DGAT1* in sheep adipocyte cultures significantly reduced the development of lipid droplets, which is in favor of the gene being involved in the metabolism and deposition of fats (Liu et al., 2023). Further evidence of the transgenic overexpression study includes the overexpression of the *SP110* gene, which showed a significant resistance to *Salmonella* infection, which has given empirical evidence of *SP110* involvement in disease resistance mechanisms (Zhou et al., 2018; Zhang et al., 2025).

## 5 Comparative analyses and discussion

Comparative genomics, both within the species (across breeds) and with other livestock, provides deeper insights into the evolution of traits and the conservation of biological pathways (Thorne et al., 2021; Ma et al., 2025).

### 5.1 Cross-breed comparisons: a world of ovine diversity

Adaptation to diverse environments and production systems results in an amazing diversity in sheep breeds. Genomic comparisons of cross-breeds show some differences in the level of selection across breeds (Fonseca et al., 2024; Gurgul et al., 2021). As an example, commercial breeds such as Merino show high levels of selection in areas related to wool and growth phenotypes such as *KRTAP* clusters and *NCAPG* (Al-Mamun et al., 2015; Sun et al., 2024). However, indigenous breeds like Tibetan sheep exhibit selective sweeps in both high-altitude hypoxia-associated genes like *EPAS1* and *UCP* genes, an oxygen metabolism gene, and an energy-generating gene, respectively (Wei et al., 2016; Ahmad et al., 2021). Tibetan sheep exhibit selective sweeps in both high-altitude hypoxia-associated genes like *EPAS1* and *UCP* genes, an oxygen metabolism gene, and an energy-generating gene, respectively. Equally, desirable alleles of parasite resistance are most likely to be associated with the regionally adapted breed, Red Maasai, but not with commercial Dorpers, which highlights the need to maintain indigenous genetic diversity as sources of resistance and adaptation to climate (Benavides et al., 2015; Silva et al., 2012). Also, convergent evolution demonstrates that similar phenotypes like prolificacy may be a result of variant genetic structures or major mutations, as with the *BMPR1* B mutation in Booroola Merinos (Zhou et al., 2018; Zhang et al., 2020). The genetic studies also showed population structure among breeds; as an example, the SNP array analysis of Polypay sheep in detail proved that there was sub-lineage stratification in breeds necessary to ensure the accuracy of GS design to prevent cryptic relatedness (Kaseja, 2023).

### 5.2 Contrasts with other livestock: the ruminant blueprint

Sheep, as ruminants, have a large part of their evolutionary history and genetic architecture in common with other livestock, especially cattle (Ibeagha-Awemu et al., 2008; Hassanine et al., 2025). Many key production traits in sheep are mediated by conserved QTL that contain orthologous interspecies genes. This can be exemplified by the *NCAPG-LCORL* locus on chromosome 6 (OAR6), which is a significant QTL in growth not only in sheep, but also in cattle and other mammals. This locus has been associated with body size phenotypes across multiple species (Takasuga, 2016; Majeres et al., 2024). Likewise, milk production QTL in sheep usually overlap genome regions that control milk phenotypes in cattle, including the casein gene cluster, to transfer knowledge to another species in the context of candidate gene prioritization (Usai et al., 2019; Zhang et al., 1998). Similar studies of functional genomics demonstrate that these genes may not only be conserved but also have common regulation. A recent cross-species DNA methylation atlas in cattle, sheep, and goats showed conservation of complex trait regulatory loci (Wadood et al., 2025). Graph-based pangenomes of ruminants are being developed in order to tackle the challenges of structural variation that are common in immune and milk protein loci (Ma et al., 2025). Through a combination of variation in different breeds and species, these frameworks are expected to increase the variant detection and cross-species transfer of QTL. These achievements indicate the strength of comparative genomics and innovative reference systems of livestock genetic enhancement (Hassanine et al., 2025; Ma et al., 2025).

## 6 Future perspectives and research gaps

Sheep genomics is a developing field at a fast rate. The next decade is likely to bring even greater transformations, as a result of new technologies, integrations between data, and new applications in breeding.

### 6.1 Novel technologies on the Horizon

Recent research is focused on increasing efficiency, minimizing mosaicism in embryos, and developing more reliable delivery systems (Tan et al., 2023; Wadood et al., 2025). It is noteworthy that the dual-gene knockout sheep of *MSTN* and *FGF5* show that it is possible to quickly assemble desirable attributes (e.g., increased muscle mass and better fleece coverage) (Kalds et al., 2022; Crispo et al., 2015). Development of more precise editing and further development in homology-directed repair are making commercial applications possible (Anzalone et al., 2019). In the meantime, lcWGS and imputation to pangenome references are rapidly becoming the method of choice for genotyping (Casellas et al., 2021; Ma et al., 2025). Another major development in livestock research is the high-throughput phenotyping with on-animal sensor technologies, which include accelerometers, GPS, and biosensors. These tools provide high-resolution, real-time observation of behavioral, physiological, and health characteristics (Berckmans, 2017). Combining these digital phenotypes with genomic data has a huge potential to choose not only complex traits but also those associated with animal welfare, social behavior, and stress resilience, as one of the bottlenecks in livestock genomics right now (Berckmans, 2017; Tan et al., 2023).

### 6.2 Data integration and analytical challenges

The future genetic discovery in livestock is based on the combination of multi-omics information, such as genomics, transcriptomics, proteomics, metabolomics, and epigenomics (Wadood et al., 2025; Ghafouri et al., 2024). Resources like the Animal GWAS Atlas are an important beginning of such integration, but one of the key challenges is still to fix the data and metadata across the studies to make the data findable, accessible, interoperable, and reusable (Tian et al., 2020). Organizations like the International Society for Animal Genetics are advancing harmonized data processing pipelines to overcome these barriers. With the expanded richness of genomic data, particularly with WGS, more advanced GS models, like Bayes RC, which use functional annotations to weight variants, machine learning, and deep learning methods, are being created to increase the accuracy and predictive ability of GS (Kaseja, 2023; Ma et al., 2025). This combination of multi-omics and sophisticated statistical models has the potential to bring precision breeding, which allows for the selection of complex traits more precisely, enhances the health of animals, and increases productivity (Hassanine et al., 2025; Wadood et al., 2025).

### 6.3 Future breeding applications and research gaps

Due to climate change, introducing new breeding challenges for resilience in sheep is becoming more important (Ramón et al., 2021; Tsartsianidou et al., 2021). The USDA Sheep GEMS project is an innovative project to produce a multi-breed GS training population in the United States that is aimed at producing increased resistance to parasites and heat tolerance. It is expected that the project will lead to the enhancement of the genomic prediction to more than 0.60 by using the data of such breeds as Katahdin, Polypay, Rambouillet, and Suffolk under the national sheep improvement program (USDA NIFA, 2022). The primary focus of breeding in the future is the discovery and incorporation of adaptive alleles in indigenous breeds into commercial populations to enhance the robustness (Fonseca et al., 2024; Thorne et al., 2021). It is essential to balance the results of selection because in most cases, QTL (e.g., SOCS2) have antagonistic pleiotropy in terms of effects on production traits and health or resilience (Rupp et al., 2015). This complexity underscores the need for whole-genome selection indexes and optimized mating plans to ensure overall genetic improvement without compromising animal fitness (Kaseja, 2023). Most QTL and candidate gene functional validation require clarification, which highlights the need to use high-throughput screening strategies coupled with specific gene editing to transition between association and causation (Tan et al., 2023; Wadood et al., 2025). These efforts together aim to harness genomics for sustainable breeding that enhances both productivity and climate resilience (Hassanine et al., 2025).

### 6.4 Economic impact analysis of genomic selection

The adoption of GS in different sheep breeding programs has increased, but there was significant variation in its implementation depending on infrastructure, data recording systems, and the nature of production systems across regions (Kaseja, 2023; Sheep Genetics Annual Outcomes Report 2023–24). Economic evaluations consistently indicate that GS can substantially improve the rate of genetic progress compared with conventional selection systems. Across breeding programs, increases in genetic gain have ranged from 25 to 40 percent depending on trait architecture, breed, and breeding objectives (Wicki et al., 2024; Oget et al., 2019; British Texel Sheep Society, 2018). This improvement translates into highly favorable Return on investment (ROI). In New Zealand, the Sheep Improvement Limited (SIL) program reported a 30 percent reduction in generation interval, accompanied by a 25 percent increase in genetic gain for meat sheep, with an estimated 250 percent ROI over a 10-year period. For growth traits in the same program, gains of 35 percent with ROI values reaching 280 percent have also been documented (Sheep Improvement Limited, 2020). In Australia, Merino breeding programs applying GS for wool quality traits achieved approximately 25 percent higher genetic gain with an estimated ROI of 220 percent (Wicki et al., 2024). In the United Kingdom, Texel breeding initiatives targeting muscle mass reported genetic gains of 40 percent and ROI values approaching 300 percent (British Texel Sheep Society, 2018). Additional benefits have also been observed in resilience and health traits. The US Sheep GEMS program reported prediction accuracies greater than 0.60 for resilience-related traits, with projected feed-cost savings of 673,651 tons annually through improved efficiency and climatic robustness (USDA NIFA, 2022). In French dairy sheep, genomic programs targeting mastitis resistance generated approximately 40 percent greater genetic gain than conventional approaches (Oget et al., 2019). In China, Hu sheep breeding programs focused on reproductive traits reported genetic improvements of around 30 percent with estimated economic returns of 190 percent (Li et al., 2024). Despite the advantages of genomic technologies, there are still some challenges that restrict wider use, particularly for health-related traits such as gastrointestinal parasite resistance, where continued genomic validation remains necessary to bring wider adoption (Eady et al., 1998; Vera et al., 2024). High-density genotyping remains costly in some production systems, and substantial computational resources are required for data processing and genomic prediction (Sharma et al., 2015). Furthermore, the availability of large and well-characterized reference populations, often exceeding 1,000 phenotyped and genotyped animals, is essential for maintaining prediction accuracy (Kaseja, 2023; Ma et al., 2025). Collaborative breeding networks and international consortia have therefore become increasingly important for aggregating phenotypic and genomic information across flocks and countries (Thorne et al., 2021). Trait complexity also affects GS efficiency. For low-heritability traits such as fertility, where heritability may be below 0.10, prediction accuracies are generally lower than for production traits. This highlights why multi-trait adopting and integrative modeling strategies should be adopted to improve the results of selection, especially when combining disease resilience with productive performance (Eady et al., 1998).

Overall, the documented success stories from New Zealand, Australia, Europe, China, and the United States demonstrate that GS can deliver major economic and biological benefits in sheep breeding systems when supported by strong infrastructure, reliable phenotyping, and sustained technical capacity. These successes underscore the potential of GS to make significant improvements in the genetic advancement in various production systems as long as it is well-supported by infrastructure and technical capacity, including breeding programs targeting footrot resistance and flock robustness (Walkom et al., 2022).

### 6.5 Emerging CRISPR applications in sheep breeding

#### 6.5.1 Established experimental evidence and proof-of-concept

The transition of CRISPR-Cas9 technology from theoretical proof-of-concept to practical application in sheep breeding marks a significant milestone in livestock genomics. Established experimental evidence demonstrates the feasibility of precisely editing major genes to enhance economically valuable traits. For instance, targeted knockouts of the *MSTN* gene have successfully produced “double-muscled” sheep with a 20%–30% increase in muscle mass and improved carcass quality without compromising meat quality (Crispo et al., 2015; Kalds et al., 2022). Similarly, CRISPR-mediated disruption of the *FGF5* gene has yielded sheep with significantly longer wool fibers, validating its role in hair cycle regulation (Kalds et al., 2022). In the realm of reproduction, precise editing of the *BMPR1B* gene to introduce the Booroola (*FecB*) mutation in Chinese Merino fine-wool sheep resulted in lambs with litter sizes up to 70% higher than wild-type controls, effectively increasing prolificacy while preserving fine wool characteristics (Zhou et al., 2018; Zhang et al., 2025). Furthermore, functional validation for disease resistance has been achieved through RNA interference and transgenic approaches, such as the knockdown of the *PRNP* to reduce prion accumulation (Sutou et al., 2007) and the overexpression of *SP110* to enhance resistance to *Salmonella* (Bishop and Morris, 2007). Regarding tail phenotypes, recent multi-omics and CRISPR-based studies have revealed a complex regulatory network underlying tail fat deposition, involving a wide array of candidate genes including *BMP2, VRTN, PDGFD,* and members of the *FABP4/PPARA* pathways (Deniskova et al., 2019). Furthermore, proof-of-concept functional validation demonstrated that CRISPR/Cas9-mediated knockout of a specific enhancer region suppressed VEGFA expression, whereas dCas9-P300-mediated activation successfully enhanced its transcription (Jin et al., 2026).

#### 6.5.2 Future potential and next-generation editing tools

Looking beyond current proof-of-concept studies, the future of CRISPR applications in sheep lies in the deployment of next-generation editing tools and multiplex strategies. Emerging technologies such as base editors and prime editors promise to introduce precise point mutations without requiring double-strand breaks, thereby significantly reducing the risk of unintended genomic alterations (Anzalone et al., 2019). Furthermore, the integration of artificial intelligence (AI) in guide RNA design is expected to optimize multiplex editing, allowing for the simultaneous improvement of multiple complex traits, such as combining enhanced muscularity with improved disease resilience or reduced methane emissions (Wadood et al., 2025). Advancements in non-viral delivery methods and somatic cell nuclear transfer (SCNT) are also anticipated to improve editing efficiencies and reduce embryonic damage associated with traditional zygote microinjection (Tan et al., 2023).

#### 6.5.3 Limitations and challenges for commercial integration

Despite these remarkable achievements, the commercial integration of gene-edited sheep faces substantial biological, regulatory, and societal limitations that must be critically addressed. Biologically, challenges such as mosaicism—where edited and unedited cells coexist within the same organism—can lead to genetic heterogeneity and unpredictable phenotypic outcomes. Furthermore, off-target mutations remain a critical concern, necessitating rigorous whole-genome sequencing to verify the specificity of the edits. From a regulatory and ethical standpoint, the classification of gene-edited animals varies globally; some jurisdictions treat them as traditional genetically modified organisms (GMOs), requiring stringent, costly, and time-consuming risk assessments. This regulatory uncertainty, coupled with concerns regarding animal welfare, biodiversity, and the high costs associated with the research and development of edited lines, poses significant barriers to commercial scalability. Finally, public acceptance and consumer perception of gene-edited livestock remain major hurdles, requiring transparent communication and robust ethical frameworks to ensure the sustainable and responsible deployment of these technologies in modern sheep breeding programs.

## 7 Conclusion and future directions

The evolution of sheep genetics over the past 3 decades reflects a significant transition from rudimentary genetic maps to high-resolution genomic resources (Thorne et al., 2021; Ma et al., 2025). This trajectory mirrors broader trends observed across other livestock species, particularly in the genomic dissection of complex reproductive and production traits (Panigrahi et al., 2024). While early studies relied on linkage mapping, the advent of high-density SNP arrays and GWAS) enabled high-resolution scans for trait-associated variants. The power of these dense-marker approaches for complex trait dissection has been further validated through comparative genomic studies across diverse taxa (Chen et al., 2022; Zhao et al., 2010). Subsequently, the arrival of whole-genome sequencing (WGS) revolutionized the field, allowing for the precise identification of causal variants and the detailed cataloging of genetic diversity (Ma et al., 2025; Wang et al., 2025). Furthermore, cross-species population-history approaches have proven instrumental in revealing adaptive signatures of environmental importance across livestock (Ward et al., 2024). Through these methodological advancements, major genes and replicated QTL—such as *NCAPG-LCORL* for growth, *BMPR1B* for reproduction, *KRTAP* clusters for wool, *LALBA* for milk, and *PRNP* for disease resistance—have been extensively characterized and integrated into marker-assisted and GS strategies (Takasuga, 2016; Bai et al., 2024; Zhou and Dong, 2013; Sutou et al., 2007). However, as highlighted in this review, a critical gap remains between statistical association and causal validation for many polygenic traits. Moving forward, the integration of multi-omics data offers the potential for system-level insights into gene function, revealing complex regulatory networks. For instance, long non-coding RNAs have emerged as a crucial regulatory layer in these systems, fine-tuning the expression of economically important traits (Guo et al., 2016; Ghafouri et al., 2024; Wadood et al., 2025).

To bridge the gap between marker discovery and biological mechanism, advanced gene-editing tools like CRISPR-Cas9, alongside microbiome-adjusted genomic prediction models, offer promising avenues to capture “missing heritability” and introduce favorable alleles with precision. Ultimately, realizing the full potential of sheep genomics will require overcoming specific emerging challenges. These include the implementation of high-throughput phenotyping technologies, the development of sophisticated analytics for complex multi-omics data, and the careful navigation of ethical and regulatory frameworks surrounding gene editing (Berckmans, 2017; Wadood et al., 2025). By addressing these limitations, the sheep genetics community can ensure that future breeding programs deliver productive, resilient, and sustainable flocks tailored to the demands of the 21st century.

## Glossary

ACACA: Acetyl-CoA carboxylase
BMP: Bone morphogenetic protein
BMPR1B: Bone morphogenetic protein receptor type 1B
CNVs: Copy number variations
CRISPR-Cas9: Clustered regularly interspaced short palindromic repeats
EPAS1: Endothelial PAS domain-containing protein 1
FASN: Fatty-acid synthase
FGF5: Fibroblast growth factor 5
GDF9: Growth differentiation factor 9
GS: Genomic Selection
GWAS: Genome-wide association studies
Indels: Insertions/deletions
KRTAPs: keratin-associated protein genes
LALBA: Alpha-lactalbumin
LCORL: Ligand Dependent Nuclear Receptor Corepressor Like
lcWGS: Low-coverage WGS
LD: Linkage disequilibrium
MAS: Marker-assisted selection
MCT1: Monocarboxylate transporter 1
MSTN: Myostatin
MTNR1A: Melatonin Receptor 1A
NCAPG: Non-SMC Codons in I Complex Subunit G
ODEs: Ordinary differential equations
PC-LDLA: Principal component-based LD-Linkage analysis
PRNP: Prion Protein
QTL: Quantitative trait loci
QTN: Quantitative trait nucleotide
RNA-Seq: RNA Sequencing
ROI: Return on investment
SLC16A1: Solute Carrier Family 16 Member 1
socs2: Suppressor of cytokine signaling-2
SVs: Structural variants
UCP: Uncoupling protein
VEGFD: Vascular Endothelial Growth Factor D
WGS: Whole genome sequencing
